# Loss of Consciousness and Cardiac Arrest as an Atypical Presentation of Tubercular Meningitis: A Case Report

**DOI:** 10.31729/jnma.7588

**Published:** 2022-07-31

**Authors:** Saroj Yadov, Santosh Acharya, Astha Thapa, Aditya Hirday, Agnimshwor Dahal

**Affiliations:** 1 Patan Academy of Health Sciences, Lagankhel, Lalitpur, Nepal; 2 Intensive Care Unit, Nidhan Hospital, Pulchowk, Lalitpur, Nepal

**Keywords:** *cardiac arrest*, *case report*, *tubercular meningitis*, *unconsciousness*

## Abstract

Tubercular meningitis is a devastating presentation of extra pulmonary tuberculosis, with fatality in each case without treatment. A 39 years male, a regular consumer of alcohol and a known case of major depressive disorder, presented with the alleged history of using an electric heater in a closed room, and presented to emergency with unconsciousness and cardiac arrest. As his neurological status didn't improve over 72 hrs, a magnetic resonance imaging brain was done which was nonconclusive. Electroencephalogram revealed diffuse right fronto-parietal seizure activity ceasing with midazolam injection, hence levetiracetam was started. Lumbar puncture revealed increased adenosine deaminase and nil white blood cells. Repeat lumbar puncture showed lymphocytic-predominant pleocytosis, elevated protein and low glucose. The patient was started on anti-tubercular therapy and an injection of dexamethasone was added. Repeat electroencephalogram didn't show any seizurelike activity. It is important to be aware of variety of presentations of tubercular meningitis. Delay in treatment leads to irreversible neurological damage and even death.

## INTRODUCTION

Tuberculosis (TB) is a major health concern in Nepal. Every day in Nepal, around 15 people lose their lives to TB.^1^ It is estimated to have 10.4 million new year TB cases each year, among them at least 1,00,000 individuals develop tubercular meningitis (TBM).^1^ Diagnosis of TBM is usually based on clinical evidence combined with imaging, lab findings and CSF findings.^2^ If left untreated, the case fatality rate is 100%. Patients usually present with non-specific symptoms like fatigue, malaise, anorexia, weight loss, fever and headache.^3^

## CASE REPORT

A 39-year-male with the alleged history of using an electric heater in a closed room presented to Emergency Department (ED) with unconsciousness and cardiac arrest. Further, his history revealed that he was bed-bound for the last 6 days, was a regular consumer of alcohol and had a known case of major depressive disorder (MDD). On examination, his GCS was 3/15. His pulse couldn't be appreciated. His breathing was gasping. He was resuscitated and reverted back to sinus rhythm. He was intubated, kept under inotropic support and shifted to ICU.

At ICU, CVC was inserted for inotropic and multiple drug requirements. He was kept under sedation, mechanical ventilation was continued and other supportive care was done.

As his neurological status didn't improve over 72 hrs, an magnetic resonance imaging (MRI) brain was done. The patient presenting with the alleged history of using an electric heater and a known case of major depressive disorder and a chronic consumer of alcohol raised the suspicion of toxic encephalopathy, hypoxic brain injury and alcoholic encephalopathy. The MRI brain result was inconclusive. This led to a diagnostic dilemma. Further, electroencelophalogram (EEG) was done which revealed diffuse right frontoparietal seizure activity ceasing with midazolam injection. Hence, an anti-epileptic levetiracetam injection (IV) of 1.5 gm stat, then 1 gm IV twice daily was started.

The patient had persistent tachycardia and hypertension for which labetalol was started after ruling out other causes of tachycardia and Hypertension. Later on, it was converted to infusion GTN due to persistent hypertension. The patient developed hypophosphatemia and hypokalemia, which were treated accordingly. The patient developed a fever on the 3rd day of their hospital stay and appropriate investigations were sent. He was started on injection of piperacillin-tazobactam prophylactically which was later changed to injection of ceftriaxone 1 gm twice daily, after reviewing the urine culture report. Chest X-ray revealed no significant findings.

In addition, a lumbar puncture done on Day 3 revealed increased ADA and nil WBC. Repeat LP on Day 4 showed tubercular meningitis-like features (total count-150, lymphocyte-90%, glucose-35, and protein-115). The patient was started on Anti-tubercular therapy (ATT)- tab isoniazid (H) 300 mg, tab rifampicin (R) 600 mg, tab pyrazinamide (Z) 1600 mg, tab ethambutol (E)1100 mg for 2 months and Tab isoniazid and tab rifampicin 600 mg for 4 months. Tab pyridoxine 40 mg once daily and injection (IV) dexamethasone were added. A repeat EEG was done on Day 6 which didn't show any seizure-like activity. The patient gradually improved and was extubated on day 13. The patient had persistent hypertension and tab prazosin and losartan were added. The patient was oriented and doing well. Hence, he was shifted to a ward.

## DISCUSSION

TBM is a lethal form of extra pulmonary tuberculosis. It is a major health concern in Nepal as its prevalence is higher. Every day in Nepal, around 15 people lose their lives to TB and over 180 people fall ill with this preventable and curable disease.^1^ It commonly presents non-specific symptoms like fatigue, malaise, anorexia, weight loss, fever and headache.^3^ Common risk factors for TBM are overcrowding, alcoholism, malnutrition, diabetes mellitus, head trauma, immunosuppression and HIV. Tubercular infection in CNS results in the granulomatous inflammatory response in meninges, cistern and parenchyma.^2^

Tubercular meningitis usually presents with non-specific symptoms like weight loss, anorexia, and fatigue behavioural change, seizures and loss of consciousness. However, in our case, the patient presented with the initial symptoms of loss of consciousness and cardiac arrest. The examination didn't reveal any neck stiffness. Further, alleged history of using an electric heater all night, has led to a variety of differential diagnosis such as carbon monoxide poisoning, hypoxic-ischemic encephalopathy and alcoholic encephalopathy. Patients presenting with atypical symptoms can lead to delay in diagnosis.

Acute alcohol intoxication can lead to cardiopulmonary arrest.^4^ In our case; the patient had an alleged history of consuming a large amount of alcohol. The patient landed in our emergency department with cardiac arrest and loss of consciousness. These presenting symptoms can lead to a delay in the diagnosis of tubercular meningitis. TB myocarditis is usually diagnosed after autopsy. However, it should be suspected in any TB patients presenting with sudden cardiac arrest.^5^ In a study the patient presented with fever, tachypnea, and cough and the patient went into cardiac arrest. It was unclear whether hypoxia led to cardiac arrest or if she had involvement of myocardium at the time of presentation that triggered cardiac arrhythmia.^5^ It is similar to our case, the patient presented with cardiac arrest. It was unclear whether it was due to alcohol intoxication collimating to hypoxia and cardiac arrest or involvement of myocardium at the time of presentation.

The patient presented with loss of consciousness and alleged history of using an electric heater. Thus an MRI brain was done, which was inconclusive. Thus, toxic encephalopathy was ruled out. MRI is an important diagnostic tool in TBM.^2^ MRI in TBM which generally shows focal enhancement in basal pial areas, particularly in the interpeduncular fossa However, in our case MRI brain was normal.

TBM results in non-specific EEG which is correlated with the severity of illness. It should be interpreted in light of clinic-radiological findings.^6^ Likewise in our case, EEG was done which revealed diffuse frontoparietal seizure activity. This finding prompted us to do further investigation.

Typical CSF findings in a patient with TBM include lymphocytic pleocytosis; low glucose level, and elevated protein.^7^ However, typical CSF findings may not be seen. Initial CSF findings were not congruent with the diagnosis of TBM in our case as well. Thus, it highlights the importance of clinical judgment and repeats CSF analysis if suspicion remains strong. The patient party reported significant improvement in the patient's health after treatment and physiotherapy. Despite his improvement, he still needs assistance for mobilization and will have to continue his ATT and physiotherapy.

Patients with TBM may present with nonspecific symptoms. In countries with a high prevalence of TB, TBM should be kept as a differential diagnosis according to clinical presentation. CSF counts may be misleading; hence, the use of multiplex polymerage chain reaction (PCR) may be helpful for early diagnosis in case of strong clinical suspicion. It is crucial for clinicians to have a TBM in differential diagnosis, especially in chronic alcoholic patients presenting complaints of loss of consciousness. MRI plays a significant role in diagnosis. However, it may show inconclusive findings. Therefore, clinical suspicion for the diagnosis of TBM should still be kept in case MRI doesn't show any pertinent findings.
